# Supplementary material for: Integrated profiling of human pancreatic cancer organoids reveals chromatin accessibility features associated with drug sensitivity
Source: Nat Commun. 2022 Apr 21;13:2169. doi: 10.1038/s41467-022-29857-6 (PMC9023604; doi:10.1038/s41467-022-29857-6)
Supplement: Supplementary file 1 — Supplementary Information [file 41467_2022_29857_MOESM1_ESM.pdf]

Supplementary Figure. 1

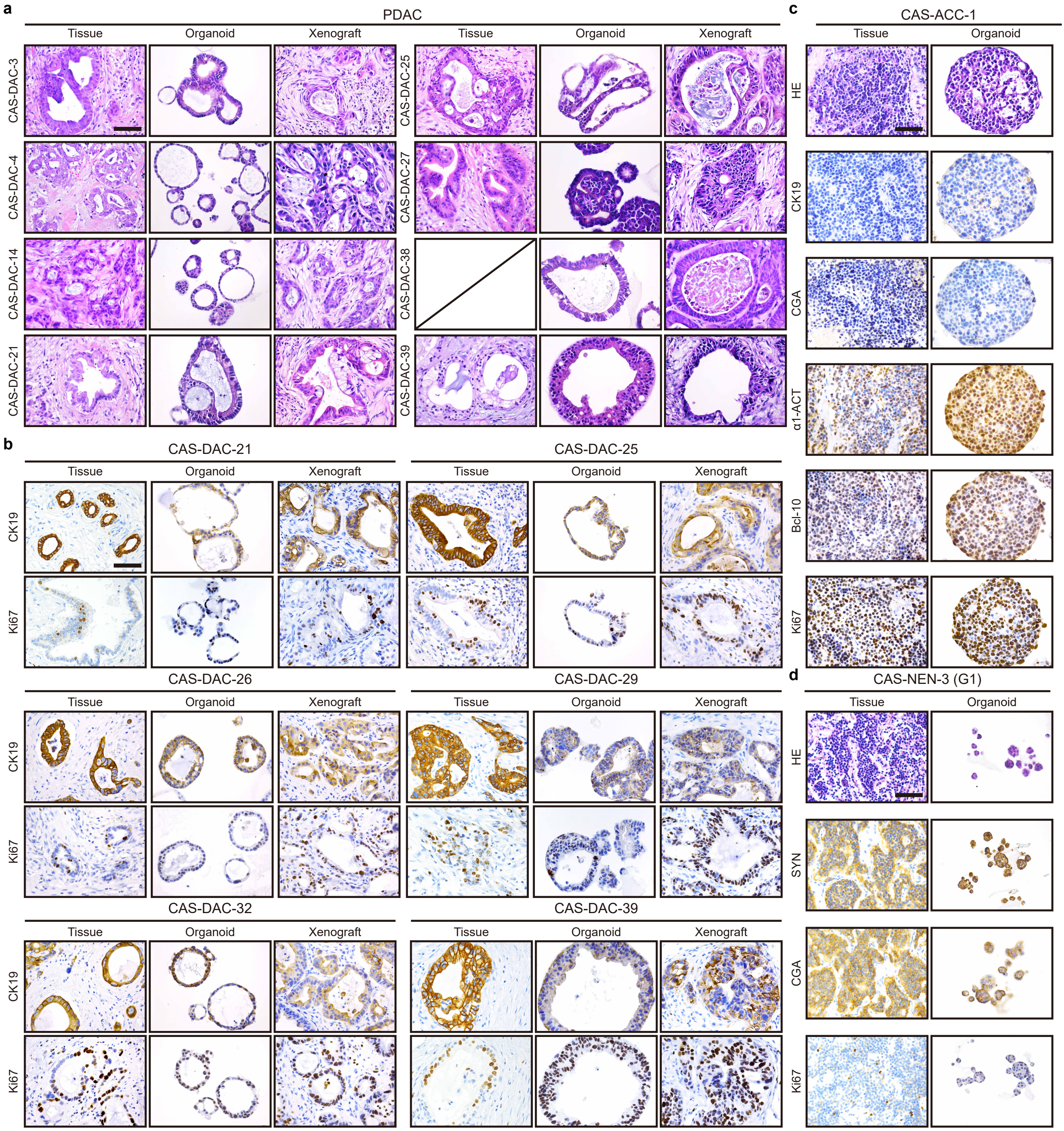

Supplementary Figure. 1 | Representative images of histological examination of PDPCOs. a, Representative H&E staining of PDAC primary tumors, organoids, and xenografts. Scale bar, 50 μm. Experiments are repeated at least three times with similar results. **b**, Representative CK19 and Ki67 staining of PDAC primary tissues, organoids and xenografts. Scale bar, 50 μm. Experiments are repeated at least three times with similar results. **c**, Representative H&E, CK19, CHGA, α1-ACT, Bcl-10 and Ki67 staining of an ACC primary tissue and organoid. Scale bar, 50 μm. Experiments are repeated at least three times with similar results. **d**, Representative H&E, SYP, CHGA and Ki67 staining of a NEN primary tissue and organoid. CAS-NEN-3 was clinically diagnosed as WHO grade G1. Scale bar, 50 μm. Experiments are repeated at least three times with similar results. PDPCO, patient-derived pancreatic cancer organoid; PDAC, pancreatic ductal adenocarcinoma; NEN, pancreatic neuroendocrine neoplasm; ACC, acinar cell carcinoma.

Supplementary Figure. 2

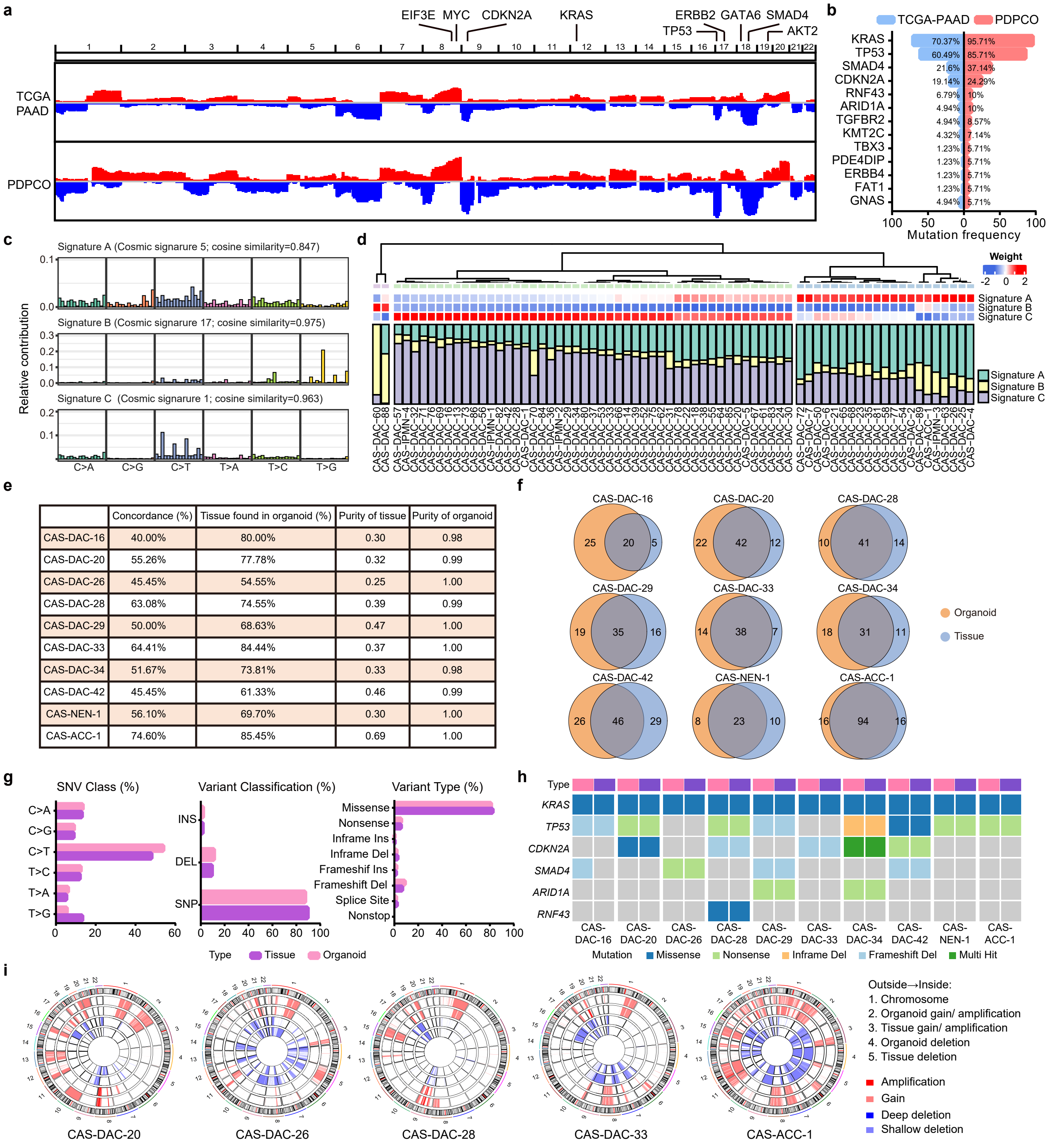

	Concordance (%)	Tissue found in organoid (%)	Purity of tissue	Purity of organoid
CAS-DAC-16	40.00%	80.00%	0.30	0.98
CAS-DAC-20	55.26%	77.78%	0.32	0.99
CAS-DAC-26	45.45%	54.55%	0.25	1.00
CAS-DAC-28	63.08%	74.55%	0.39	0.99
CAS-DAC-29	50.00%	68.63%	0.47	1.00
CAS-DAC-33	64.41%	84.44%	0.37	1.00
CAS-DAC-34	51.67%	73.81%	0.33	0.98
CAS-DAC-42	45.45%	61.33%	0.46	0.99
CAS-NEN-1	56.10%	69.70%	0.30	1.00
CAS-ACC-1	74.60%	85.45%	0.69	1.00

Supplementary Figure. 2 | Comparison of genomic alterations among exocrine PDPCOs. a, Chromosomal changes in the TCGA PAAD cohort (top) and 70 exocrine PDPCOs (bottom) visualized with Integrated Genomics Viewer. The percentage of PDPCOs with log2(copy number/2)>0.1 is shown in red, and the percentage of PDPCOs with log2(copy number/2)<-0.1 is shown in blue. **b**, Comparison of the detailed mutation frequency of common pancreatic cancer-related genes between TCGA PAAD dataset and our PDPCOs. **c**, Mutational signatures identified by the NMF method in the 70 exocrine PDPCOs. **d**, Mutational signature-based clustering of the 70 exocrine PDPCOs. Heatmap and barplot show relative contribution of three NMF mutational signatures in each sample. **e**, Purity, concordance and percent of the primary tumor mutations found in the organoid cultures among exon regions of 10 paired exocrine PDPCOs (WGS) and original tissues (WES). **f**, Representative Venn diagrams are shown of 9 paired exocrine PDPCOs and original tissues based on mutations in exon regions. **g**, Distribution of variation within the coding region of 10 paired exocrine PDPCOs and original tissues. The proportions of SNV class (left), variant classification (middle) and variant type (right) are shown. **h**, Mutation profile of the six top mutated pancreatic cancer-related genes in 10 paired exocrine PDPCOs and original tissues. **i**, Circos plot showing the comparison of copy number variants in 5 paired exocrine PDPCOs and tissues. PDPCO, patient-derived pancreatic cancer organoid; NMF, nonnegative matrix factorization.

Supplementary Figure. 3

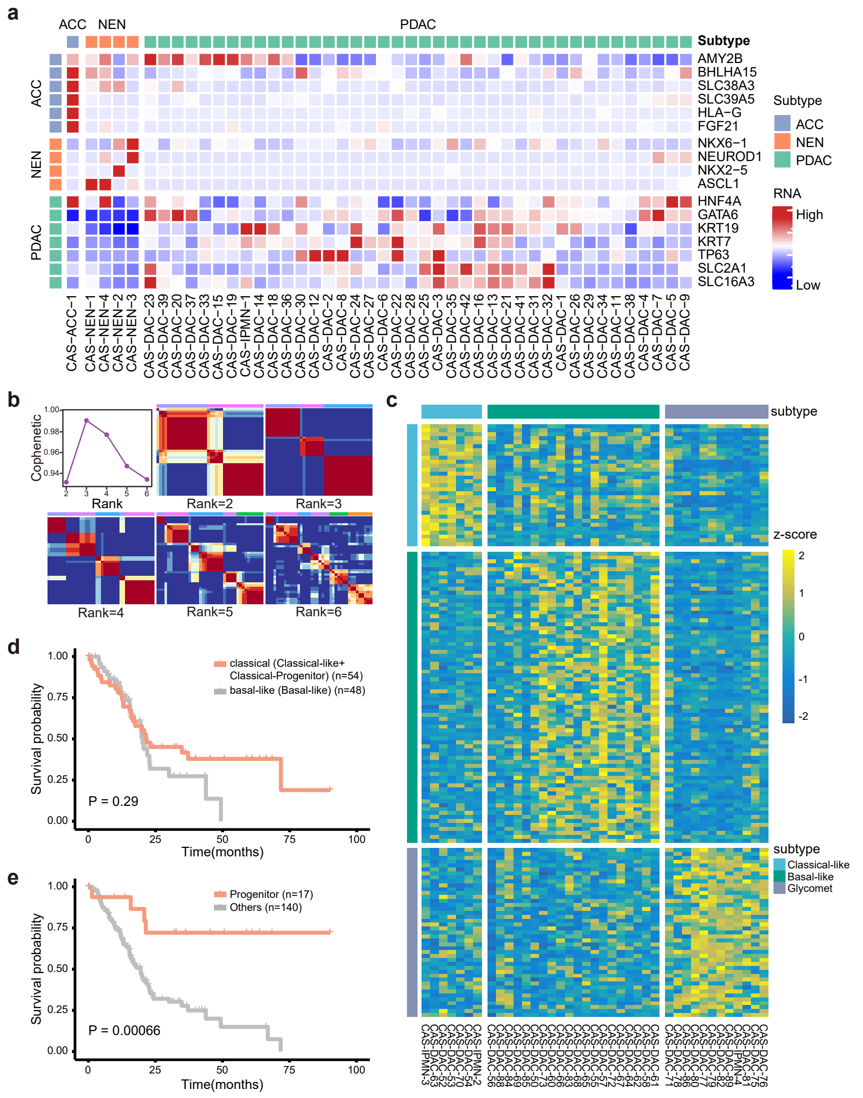

Supplementary Figure. 3 | Transcriptome-based classification of exocrine PDPCOs and validation in the internal cohort. a, Heatmap showing the expression of representative genes of acinar cell, neuroendocrine cell and PDAC in the 45 PDPCOs (39 CAS-DACs, 1 CAS-IPMN, 1 CAS-ACC and 4 CAS-NENs). **b**, Detailed data from unsupervised NMF clustering in the discovery cohort of 40 exocrine PDPCOs (39 CAS-DACs and 1 CAS-IPMN). Solutions are shown for classes k = 2 to k = 6. **c**, Validation of the transcriptomic subtypes in an internal cohort of 39 exocrine PDPCOs (36 CAS-DACs and 3 CAS-IPMNs). Samples are listed in rows, and signature genes are listed in columns as z-scores. **d**, Kaplan-Meier survival analysis showing the overall survival outcomes of patients with the classical (classical-like and classical-progenitor) and basal-like (basal-like) subtypes, as identified in Fig. 3f. P value is determined by using log-rank test. **e**, Kaplan-Meier survival analysis showing the overall survival outcomes of patients with the classical-progenitor and other (classical-like, basal-like and glycomet) subtypes, as identified in Fig. 3f. P value is determined by using log-rank test. PDPCO, patient-derived pancreatic cancer organoid; PDAC, pancreatic ductal adenocarcinoma. NEN, pancreatic neuroendocrine neoplasm; ACC, acinar cell carcinoma.

Supplementary Figure. 4

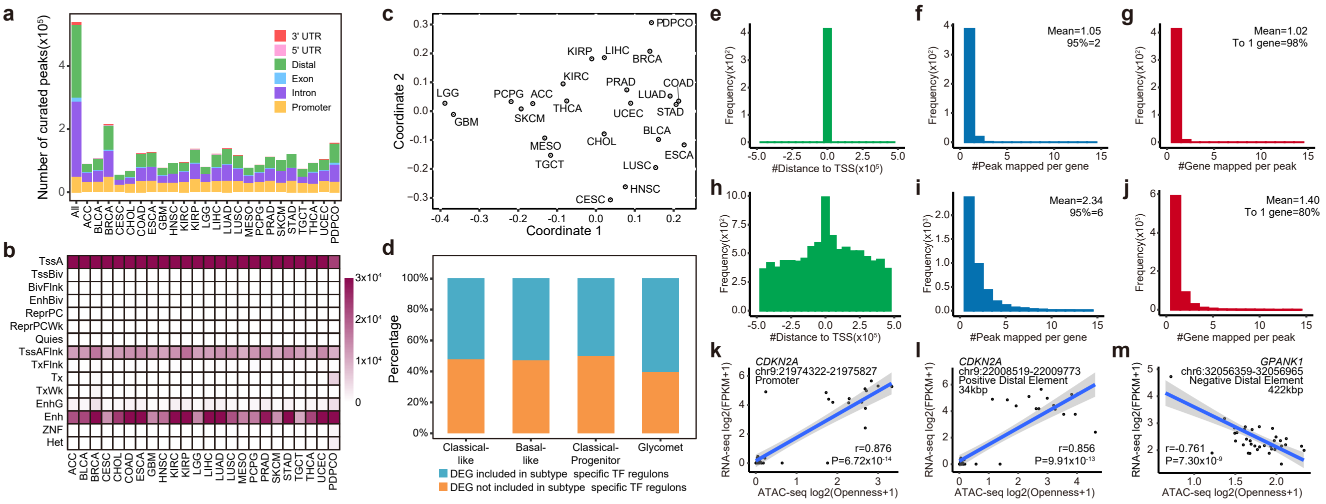

Supplementary Figure. 4 | Comparison of ATAC-seq data from PDPCOs with data from a previous pancancer study and characterization of putative peak-to-gene links. a, Reproducible peaks from ATAC-seq of 45 PDPCOs (41 exocrine and 4 neuroendocrine PDPCOs) and a pancancer dataset. Types of genomic regions with ATAC-seq peaks are labeled with different colors. **b**, Heatmap showing the enrichment of ATAC-seq peaks within ChIP-seq-defined chromHMM states from the Roadmap Epigenomics Project in above PDPCOs and pancancer dataset. Cancer types are shown at bottom. ChIP-seq-defined chromHMM states are shown at left. **c**, Principal coordinates analysis of reproducible peaks in above PDPCOs and pancancer dataset. Each dot represents a single cancer type. Cancer types are shown at bottom. **d**, Bar plot showing the proportion of DEGs covered by enriched TF regulons in each transcriptomic subtype. **e**, Distribution of the distance from each peak to the transcription start site of linked gene among promoter-to-gene links. Distribution of the number of peaks linked per gene in **f** and genes linked per peak in **g** among promoter-to-gene links. **h**, Distribution of the distance from each peak to the transcription start site of linked gene among distal peak-to-gene links. Distribution of the number of peaks linked per gene in **i** and genes linked per peak in **j** among distal peak-to-gene links. Dot plot of a representative promoter-to-gene link in **k**, a representative positive distal peak-to-gene link in **l**, a representative negative distal peak-to-gene link in **m**. Each dot represents an individual sample. P values associated with Pearson's correlation coefficients were adjusted for multiple testing using FDR. PDPCO, patient-derived pancreatic cancer organoid; DEG, differentially expressed gene. ACC, adrenocortical carcinoma; BLCA, bladder urothelial carcinoma; BRCA, breast invasive carcinoma; CESC, cervical squamous cell carcinoma; CHOL, cholangiocarcinoma; COAD, colon adenocarcinoma; ESCA, esophageal carcinoma; GBM, glioblastoma multiforme; HNSC, head and neck squamous cell carcinoma; KIRC, kidney renal clear cell carcinoma; KIRP, kidney renal papillary cell carcinoma; LGG, low-grade glioma; LIHC, liver hepatocellular carcinoma; LUAD, lung adenocarcinoma; LUSC, lung squamous cell carcinoma; MESO, mesothelioma; PCPG, pheochromocytoma and paraganglioma; PRAD, prostate adenocarcinoma; SKCM, skin cutaneous melanoma; STAD, stomach adenocarcinoma; TGCT, testicular germ cell tumors; THCA, thyroid carcinoma; UCEC, uterine corpus endometrial carcinoma. TssA, active TSS; TssBiv, bivalent/poised TSS; EnhBiv, bivalent enhancer; ReprPC, repressed polycomb; ReprPCWk, weak repressed polycomb; Quies, quiescent/low; TssAFlnk, flanking active TSS; TxFlnk, transcription at the 5' and 3' ends of genes; Tx, strong transcription; TxWk, weak transcription; EnhG, genic enhancer; Enh, enhancer; ZNF/Rpts, ZNF genes and repeats; Het, heterochromatin.

Supplementary Figure. 5

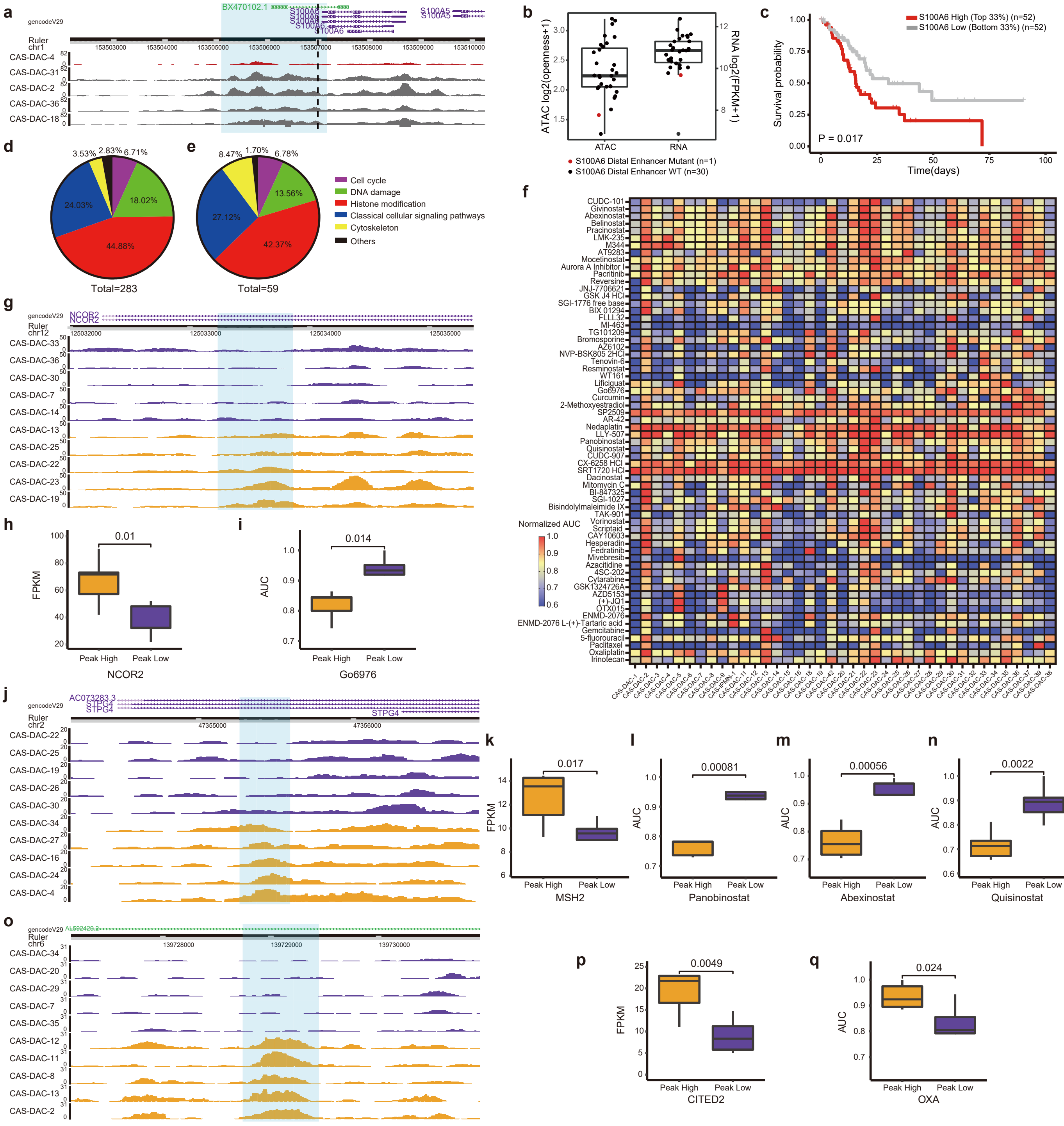

Supplementary Figure. 5 | See next page for caption.

Supplementary Figure. 5 | Integrated analysis of noncoding mutations and drug sensitivity in PDPCOs. a, Normalized ATAC-seq tracks of *S100A6* putative enhancer locus in 5 representative samples. The red and gray tracks represent samples with and without mutation of *S100A6* putative enhancer, respectively. Black dotted line indicates the position of mutation, and the predicted enhancer region is highlighted by light blue shading. **b**, Box plot showing chromatin accessibility at *S100A6* putative enhancer and *S100A6* gene expression across 31 exocrine PDPCOs. Boxplot show the median (central line), the 25–75% interquartile range (box limits). **c**, Kaplan-Meier analysis of overall survival in TCGA PAAD cohort stratified by high and low expression of *S100A6* gene. P value is determined by using log-rank test. **d**, Pie charts showing pathway classification of 283 chemicals used for primary high-throughput drug screening. **e**, Pie charts showing pathway classification of 59 chemicals used for secondary high-throughput drug screening. **f**, Heatmap of secondary high-throughput drug screening results showing AUC values of 59 chemicals and 5 chemotherapeutic agents in 39 exocrine PDPCOs. **g**, Normalized ATAC-seq tracks of *NCOR2* putative enhancer locus in 10 representative samples. The yellow and blue tracks represent samples with and without peaks in *NCOR2* putative enhancer, respectively. The peak region is highlighted by light blue shading. Comparison between peak high group with peaks in *NCOR2* putative enhancer and peak low group without such peaks in above 10 samples of *NCOR2* gene expression in **h** and AUC of Go6976 in **i**. Each group contains 5 biologically independent samples. Boxplot show the median (central line), the 25–75% interquartile range (box limits). Significance is computed by two-sided unpaired *t* test. **j**, Normalized ATAC-seq tracks of *MSH2* putative enhancer locus in 10 representative samples. The yellow and blue tracks represent samples with and without peaks in *MSH2* putative enhancer, respectively. The peak region is highlighted by light blue shading. Comparison between peak high group with peaks in *MSH2* putative enhancer and peak low group without such peaks in above 10 samples of *MSH2* gene expression in **k**, AUC of panobinostat in **l**, AUC of abexinostat in **m**, and AUC of quisinostat in **n**. Each group contains 5 biologically independent samples. Boxplot show the median (central line), the 25–75% interquartile range (box limits). Significance is computed by two-sided unpaired *t* test. **o**, Normalized ATAC-seq tracks of *CITED2* putative enhancer locus in 10 representative samples. The yellow and blue tracks represent the samples with and without peaks in *CITED2* putative enhancers, respectively. The peak region is highlighted by light blue shading. Comparison between peak high group with peaks in *CITED2* putative enhancer and peak low group without such peaks in above 10 samples of *CITED2* gene expression in **p** and AUC of OXA in **q**. Boxplot show the median (central line), the 25–75% interquartile range (box limits). Significance is computed by two-sided unpaired *t* test. PDPCO, patient-derived pancreatic cancer organoid; AUC, area under curve; OXA, oxaliplatin.

Supplementary Figure. 6

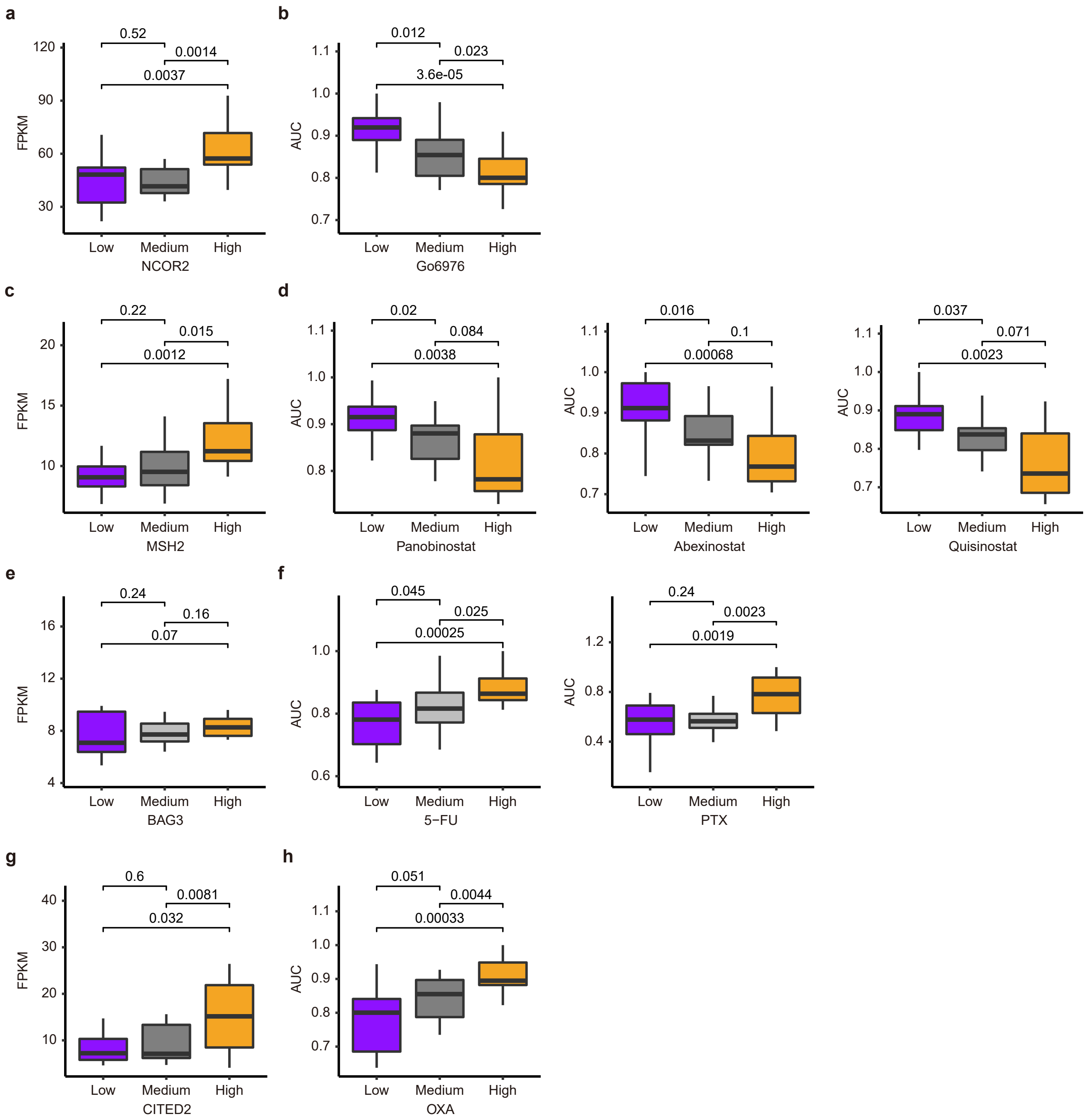

Supplementary Figure. 6 | Comparison of gene expression and drug sensitivity based on tertiles of ATAC-seq peak openness over all samples. Stratifying the openness of ATAC-peak at chr12:125033345-125034019 into tertiles over all samles (low, medium and high) to compare the expression of *NCOR2* gene in **a**, and the drug sensitivity of Go6976 in **b**. Boxplot show the median (central line), the 25–75% interquartile range (box limits). Significance is computed by two-sided unpaired *t* test. Stratifying the openness of ATAC-peak at chr2:47355361-47355625 into tertiles over all samles (low, medium and high) to compare the expression of *MSH2* gene in **c**, and the drug sensitivity of panobinostat, abexinostat and quisinostat in **d**. Boxplot show the median (central line), the 25–75% interquartile range (box limits). Significance is computed by two-sided unpaired *t* test. Stratifying the openness of ATAC-peak at chr10:121441617-121442244 into tertiles over all samles (low, medium and high) to compare the expression of *BAG3* gene in **e**, and the drug sensitivity of 5-FU and PTX in **f**. Boxplot show the median (central line), the 25–75% interquartile range (box limits). Significance is computed by two-sided unpaired *t* test. Stratifying the openness of ATAC-peak at chr6:139728666-139729424 into tertiles over all samles (low, medium and high) to compare the expression of *CITED2* gene in **g**, and the drug sensitivity of OXA in **h**. Boxplot show the median (central line), the 25–75% interquartile range (box limits). Significance is computed by two-sided unpaired *t* test.5-FU, 5-fluorouracil; PTX, paclitaxel.

Supplementary Figure. 7

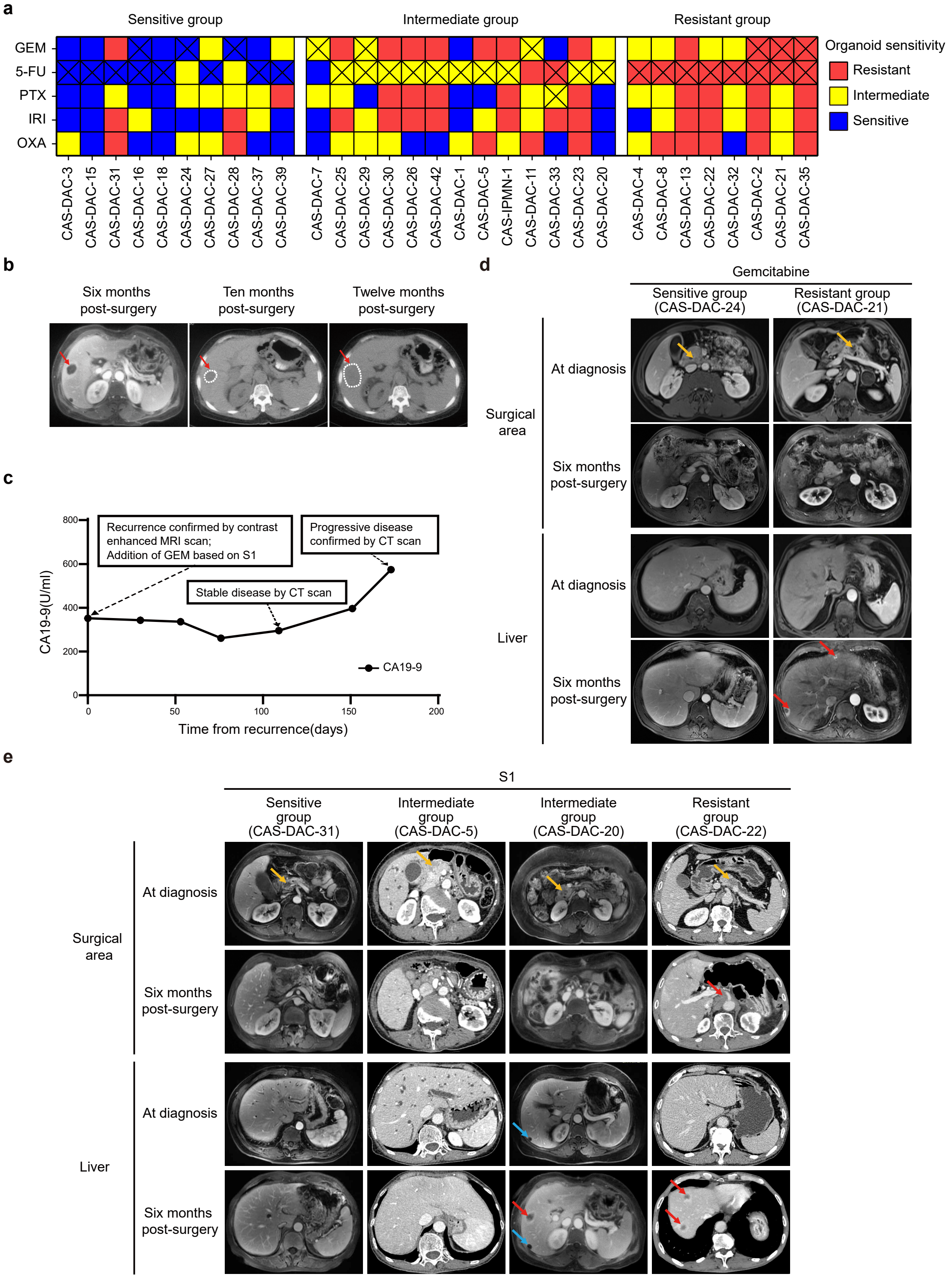

Supplementary Figure. 7 | Clinically relevant chemosensitivity of PDPCOs. a, Graphical summary of group definition and chemotherapy administration in 31 patients who underwent upfront radical surgery with subsequent adjuvant therapy. Symbol X indicated the chemotherapy agents used in clinical practice of corresponding patients. **b**, Radiation examination of the liver of patient CAS-DAC-20 at six months post surgery (CE-MRI), ten months post surgery (CT) and twelve months post surgery (CT). Red arrow, metastasis. **c**, Curve showing the levels of the tumor marker CA19-9 in patient CAS-DAC-20 after the confirmation of recurrence. Source data are provided as a Source Data file. d, Radiation examination of both surgical area and liver at the time of diagnosis and six months post-surgery in patients (CAS-DAC-24, CE-MRI; CAS-DAC-21, CE-MRI) treated with gemcitabine from different sensitivity group. Yellow arrow, primary tumor; Red arrow, metastasis or local recurrence. **e**, Radiation examination of both surgical area and liver at the time of diagnosis and six months post-surgery in patients (CAS-DAC-31, CE-MRI; CAS-DAC-5, CE-CT; CAS-DAC-20, CE-MRI; CAS-DAC-22, CE-CT) treated with S1 from different sensitivity group. Yellow arrow, primary tumor; Red arrow, metastasis or local recurrence. S1 exhibits the same mode of action as 5-FU *in vivo*. PDPCO, patient-derived pancreatic cancer organoid; GEM, gemcitabine; 5-FU, 5-fluorouracil; PTX, paclitaxel; OXA, oxaliplatin; IRI, irinotecan; CE-MRI: contrast-enhanced magnetic resonance imaging; CT: computed tomography; CE-CT: contrast-enhanced computed tomography.

Supplementary Figure. 8

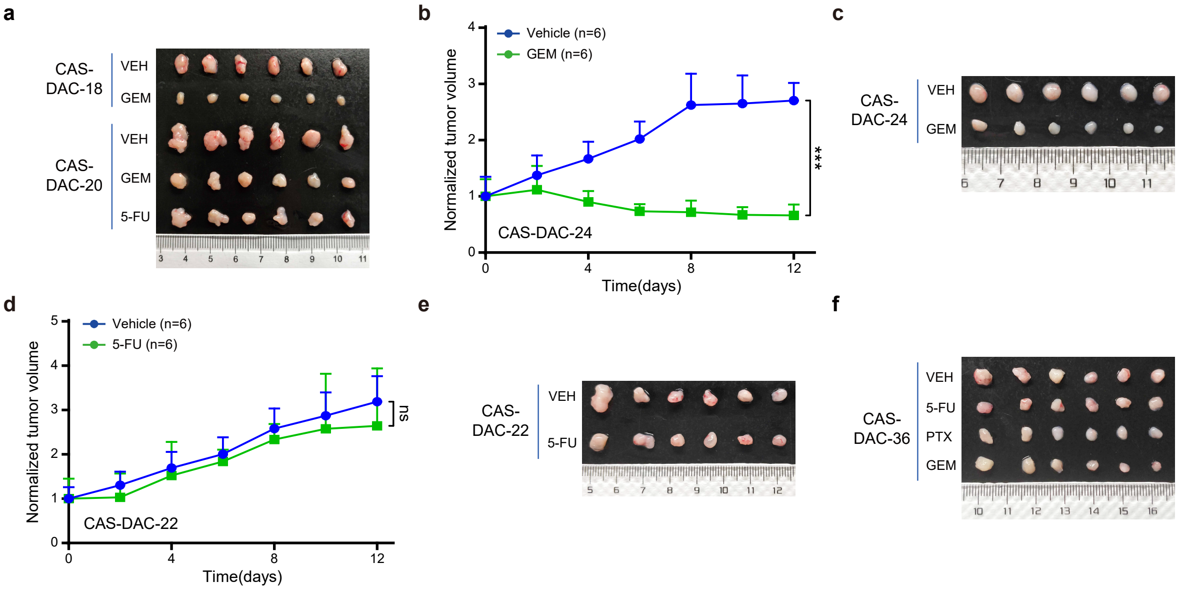

Supplementary Figure. 8 | Validation of clinically relevant chemosensitivity of PDPCOs by in vivo xenograft models. a, Top: Isolated ODX-18 tumor treated with Vehicle (n=6) and GEM (n=6). Bottom: Isolated ODX-20 tumor treated with Vehicle (n=6), GEM (n=6) and 5-FU (n=6). b, Drug test of ODX-24 with GEM (n=6), using Vehicle as a control (n=6). Data are presented as mean values + SEM. Statistical Significance was computed by two-sided unpaired *t* test. Source data are provided as a Source Data file. c, Isolated ODX-24 tumor treated with Vehicle (n=6) and GEM (n=6). d, Drug test of ODX-22 with 5-FU (n=6), using Vehicle as a control (n=6). Data are presented as mean values + SEM. Statistical Significance was computed by two-sided unpaired *t* test. Source data are provided as a Source Data file. e, Isolated ODX-22 tumor treated with Vehicle (n=6) and 5-FU (n=6). f, Isolated ODX-36 tumor treated with Vehicle (n=6), 5-FU (n=6), PTX (n=6) and GEM (n=6). Statistical analysis, ns P≥0.05,* P<0.05, ** P<0.01, *** P<0.001. PDPCO, patient-derived pancreatic cancer organoid; GEM, gemcitabine; 5-FU, 5-fluorouracil; PTX, paclitaxel; VEH, vehicle.

Supplementary Figure. 9

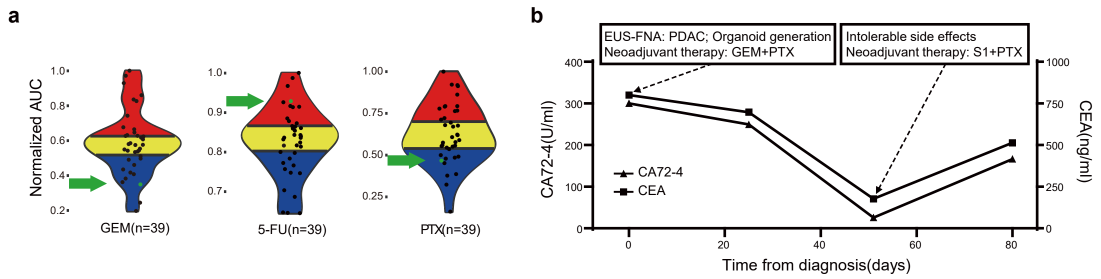

Supplementary Figure. 9 | Clinically relevant chemosensitivity of PDPCOs in neoadjuvant therapy setting. a, Chemosensitivity of organoid CAS-DAC-38 (green dot indicated by the green arrow) to GEM, 5-FU and PTX. **b**, Curves showing the levels of the tumor markers CA72-4 and CEA in patient CAS-DAC-38 after pathological diagnosis of PDAC by EUS-FNA. S1 exhibits the same mode of action as 5-FU *in vivo*. Source data are provided as a Source Data file. PDPCO, patient-derived pancreatic cancer organoid; GEM, gemcitabine; 5-FU, 5-fluorouracil; PTX, paclitaxel; EUS-FNA, endoscopic ultrasound-guided fine needle aspiration biopsy.
