# Supplementary material for: Integrated profiling of human pancreatic cancer organoids reveals chromatin accessibility features associated with drug sensitivity
Source: Nat Commun. 2022 Apr 21;13:2169. doi: 10.1038/s41467-022-29857-6 (PMC9023604; doi:10.1038/s41467-022-29857-6)
Supplement: Supplementary file 3 — Description of Additional Supplementary Files [file 41467_2022_29857_MOESM3_ESM.pdf]

Description of Additional Supplementary Files

File Name: Supplementary Data 1
Description: Clinicopathologic variables and sample sequencing for the PDPCO biobank.

File Name: Supplementary Data 2
Description: Coding and noncoding mutations in the 69 exocrine PDPCOs with paired organoid and blood WGS.

File Name: Supplementary Data 3
Description: DEGs of four transcriptomic subtypes in the 40 exocrine PDPCOs and DEGs of validated subtypes in the 39 exocrine PDPCOs.

File Name: Supplementary Data 4
Description: The 283 TF regulons available for motif enrichment analysis and the enriched TF regulons in NEN as well as PDAC subtypes among 45 PDPCOs.

File Name: Supplementary Data 5
Description: Prediction of links between ATAC-seq peak and targeted gene expression in the 41 exocrine PDPCOs.

File Name: Supplementary Data 6
Description: Noncoding mutations within ATAC-seq peaks among the 31 exocrine PDPCOs.

File Name: Supplementary Data 7
Description: Chemicals and chemotherapy used in primary and secondary high throughput drug screening in the exocrine PDPCOs.

File Name: Supplementary Data 8
Description: Normalized AUC of drug screening in 39 exocrine PDPCOs with 59 chemical and 5 chemotherapeutic drugs.

File Name: Supplementary Data 9
Description: Prediction of links between ATAC-seq peak and drug sensitivity in the 39 exocrine PDPCOs.

File Name: Supplementary Data 10
Description: Clinical follow-up data for the 39 matched patients from the prospective database of Changhai Hospital.

File Name: Supplementary Data 11
Description: Detailed information of chemicals, peptides, and recombinant proteins involved in this study.
